# Follicle-Stimulating Hormone Induces Lipid Droplets *via* Gαi/o and β-Arrestin in an Endometrial Cancer Cell Line

**DOI:** 10.3389/fendo.2021.798866

**Published:** 2022-02-03

**Authors:** Niamh S. Sayers, Priyanka Anujan, Henry N. Yu, Stephen S. Palmer, Jaya Nautiyal, Stephen Franks, Aylin C. Hanyaloglu

**Affiliations:** ^1^ Department of Metabolism, Digestion and Reproduction, Institute of Reproductive and Developmental Biology, Imperial College London, London, United Kingdom; ^2^ CanWell Pharma Inc., Wellesley, MA, United States; ^3^ Department of Pathology and Immunology, Baylor College of Medicine, Houston, TX, United States; ^4^ Department of Surgery and Cancer, Imperial College London, London, United Kingdom

**Keywords:** FSH, FSHR, endometrial cancer, Ishikawa cells, arrestin, G protein, GPCR

## Abstract

Follicle-stimulating hormone (FSH) and its G protein-coupled receptor, FSHR, represents a paradigm for receptor signaling systems that activate multiple and complex pathways. Classically, FSHR activates Gαs to increase intracellular levels of cAMP, but its ability to activate other G proteins, and β-arrestin-mediated signaling is well documented in many different cell systems. The pleiotropic signal capacity of FSHR offers a mechanism for how FSH drives multiple and dynamic downstream functions in both gonadal and non-gonadal cell types, including distinct diseases, and how signal bias may be achieved at a pharmacological and cell system-specific manner. In this study, we identify an additional mechanism of FSH-mediated signaling and downstream function in the endometrial adenocarcinoma Ishikawa cell line. While FSH did not induce increases in cAMP levels, this hormone potently activated pertussis toxin sensitive Gαi/o signaling. A selective allosteric FSHR ligand, B3, also activated Gαi/o signaling in these cells, supporting a role for receptor-mediated activation despite the low levels of *FSHR* mRNA. The low expression levels may attribute to the lack of Gαs/cAMP signaling as increasing FSHR expression resulted in FSH-mediated activation of the Gαs pathway. Unlike prior reports for FSH-mediated Gαs/cAMP signaling, FSH-mediated Gαi/o signaling was not affected by inhibition of dynamin-dependent receptor internalization. While chronic FSH did not alter cell viability, FSH was able to increase lipid droplet size. The β-arrestins are key adaptor proteins known to regulate FSHR signaling. Indeed, a rapid, FSH-dependent increase in interactions between β-arrestin1 and Gαi1 was observed *via* NanoBiT complementation in Ishikawa cells. Furthermore, both inhibition of Gαi/o signaling and siRNA knockdown of β-arrestin 1/2 significantly reduced FSH-induced lipid droplet accumulation, implying a role for a Gαi/o/β-arrestin complex in FSH functions in this cell type. As FSH/FSHR has been implicated in distinct hormone-dependent cancers, including endometrial cancer, analysis of the cancer genome database from 575 human endometrial adenocarcinoma tumors revealed that a subpopulation of samples expressed FSHR. Overall, this study highlights a novel mechanism for FSHR signal pleiotropy that may be exploited for future personalized therapeutic approaches.

## Introduction

The gonadotropin hormone, follicle-stimulating hormone (FSH) is secreted from the anterior pituitary and plays key role in reproduction *via* activation of its receptor, FSHR in the gonads. In females, FSH-mediated activation of its receptor expressed in ovarian granulosa cells, induces steroidogenesis and stimulates growth of the follicle. While in males, FSH activation of FSHR in testicular Sertoli cells regulates spermatogenesis (1).

FSHR belongs to the Class A/Rhodopsin subfamily of the largest class of membrane proteins, the G protein-coupled receptors (GPCRs). GPCRs represent a substantial portion of current FDA-approved drug targets (2) and their success as a drug target is in part due to their significant representation in the human genome, their widespread expression and their central roles in all physiological systems including many diseases such as cancer (3–6). GPCRs transduce extracellular signals into intracellular cascades through four main heterotrimeric G protein pathways: Gαs, Gαi/o, Gαq/11, and Gα12/13, each activating a specific and complex downstream signaling pathway. In addition, many receptors recruit a member of the key GPCR scaffolding protein family, the arrestins/β-arrestins (the non-visual arrestins comprised of β-arrestin 1 and β-arrestin 2) following receptor activation and phosphorylation. The β-arrestins play numerous roles in regulating GPCR activity in part due to its ability to adopt multiple distinct conformations in complex with the receptor (7–11), although its ability to associate with other signal proteins, including G proteins, have also been reported (12–17). Thus, in addition to classical roles in G protein-uncoupling and receptor internalization, β-arrestins can form signalosomes that interact with multiple partners and induce diverse downstream signaling pathways.

To achieve the multiple functions that GPCRs play *in vivo*, many receptors adopt several mechanisms to achieve signal diversity, including, but not limited to; coupling to multiple G proteins, association with distinct GPCRs as heteromers and the ability to activate signaling not only from the plasma membrane, but also from distinct intracellular compartments (18–20). The FSHR is a good example of a GPCR that employs all of these mechanisms to diversify its signaling. Classically, FSHR activates Gαs to increase intracellular levels of cAMP, but its ability to activate Gαq/11 and in particular Gαi/o is well documented in distinct cell types. FSH/FSHR signaling is known to recruit the β-arrestins for its trafficking, signaling to MAPK pathways but also FSHR-mediated translation *via* a β-arrestin/p70S6K/ribosomal S6 complex (21–24). FSHR can also interact as heteromers with distinct GPCRs to modulate its signaling (25–27) and we have recently demonstrated that this receptor requires internalization to fully activate Gαs signaling from specific intracellular compartments termed very early endosomes (VEE) (28, 29). This complexity in signal profiles offers the ability of GPCRs such as FSHR to exhibit bias in their signaling, where one, or more, pathways, may be preferentially activated over others. Such bias has been well documented and may be both ligand and cell/system specific (30–34). The pluri-dimensional signaling capacity of FSHR offers a mechanism underlying the multiple and dynamic functions of FSH/FSHR in target cells including proliferation, steroidogenesis, apoptosis and differentiation (31, 35–37). These and other functions of FSH may not be restricted to the gonads as there have been increasing reports of FSHR expression and function in extragonadal sites including, bone, adipose, uterine endometrium and myometrium and umbilical endothelial cells (30, 32, 38–43). While the expression and functions of extragondal FSH/FSHR has been debated (44), the role of this receptor in distinct diseases has also emerged including endometriosis, and several cancers including ovarian cancer, breast cancer, prostate cancer and endometrial cancer (45–54), which could exploit the multiple pathways this receptor can activate and/or represent an unexploited target in disease. The incidence and death rates of certain cancers such as endometrial adenocarcinoma are increasing (55). Factors pre-disposing women to this intricately hormone-linked cancer include nulliparity, early menarche, late menopause, obesity, diabetes, hypertension, polycystic ovarian syndrome (PCOS), sub-fertility and estrogen therapy, prescribed to relieve the symptoms of menopause. Thus, the age group with the highest incidence of endometrial cancer is in post-menopausal women, where circulating serum FSH levels are also raised. FSHR expression has been linked to angiogenic activities in epithelial and endothelial cells of the endometrium (56) and reported to be localized to endothelial cells of cancer vasculature (57), although these initial reports with distinct FSHR antibodies were not confirmed in a key follow-up study (58). However, direct functional effects of FSH in endometrial cancer cell lines have indicated potential pro-viability functions although *via* Gαs/cAMP-independent pathways (47). Given that our understanding of GPCR signaling has evolved rapidly in the past few years, these pathophysiological systems may provide further insight into how cells may ‘rewire’ signal systems in disease.

In this study, we employed endometrial adenocarcinoma cells to assess whether FSH/FSHR could activate multiple pathways, and if such activity is regulated at a spatial-temporal level that we and others have demonstrated in heterologous cell lines. We demonstrate that the low expression levels of FSHR in Ishikawa cells do not increase levels of cAMP upon FSH treatment, yet potently activates Gαi/o signaling. This G protein signaling occurred *via* an internalization-independent, but β-arrestin-dependent mechanism, whereby FSH promotes complex formation of Gαi/β-arrestin in these cells. Unexpectedly, we identify a role for FSH-mediated activation of Gαi/β-arrestin in driving lipid droplet enlargement. These findings provide new insights into the manifold activities of FSH/FSHR, including the role of arrestins in FSHR signaling and potential avenues of future investigation for pathophysiologies involving this increasingly complex hormone receptor system.

## Materials and Methods

### Reagents

Human pituitary FSH (A.F. Parlow, National Hormone and Peptide Program, Harbor-UCLA Medical Center, purity ≥95%) was diluted in PBS and stored at -20°C then diluted in MEM at appropriate concentrations. A previously characterized FSHR low molecular weight ligand (B3; TocopheRx, Burlington, MA, USA) was employed (29, 59). Purity of B3 ranged from 95-97%. Ligands were diluted in sterile dimethyl sulfoxide (DMSO, Sigma Aldrich) and stored at +4°C then diluted in MEM at appropriate concentrations. The antibodies used were mouse anti-FLAG (Sigma-Aldrich) and AlexaFluor 555 goat anti-mouse IgG (Invitrogen), rabbit anti-β-Arrestin1/2 (Cell Signaling), mouse anti-GAPDH (EMD Milipore), horseradish Peroxidase (HRP)-conjugated goat anti-rabbit IgG (Santa Cruz) and HRP-conjugated goat anti-mouse IgG (Santa Cruz). The dynamin GTPase inhibitor, dyngo-4a was purchased from Abcam. Furimazine (Promega) was used as the substrate for NanoLuc. IBMX (Sigma-Aldrich) was used to inhibit the degradation of intracellular cAMP and forskolin (Cayman) to directly activate adenylate cyclase. Pertussis toxin (Tocris) was used to inhibit Gαi/o activation. Knockout Serum Replacement (KSR; Gibco) was used to induce lipid droplet accumulation. BODIPY 493/503 (ThermoFisher Scientific) was used to dye neutral lipids. PGE_2_ and isoproterenol were both purchased from Sigma.

FLAG-hFSHR has been previously described (28). SmBiT-β-Arrestin 1 and LgBiT-Gαi1 were kindly provided by Dr Asuka Inoue (Tohoku University). siRNA to knockdown β-arrestin1/2 was obtained from ThermoFisher Scientific; β-Arrestin 1 (HSS180974) and β-Arrestin 2 (HSS180982).

Primers used in this study were as follows: FSHR (forward 5’: CCCTGCTCCTGGTCTCTTTG; reverse 5’: CTCGAAGCTTGGTGAGGACA). FSHR Exon 9 deletion (forward 5’: GGACCAGTCATTCTCTCTGAGC; reverse 5’: CTTCATTGCATAAGTCATAGTC). β-Actin (forward 5’: CTCTTCCAGCCTTCCTTCCT; reverse 5’: AGCACTGTGTTGGCGTACAG).

### Cell Culture and Transfection

Ishikawa human endometrial adenocarcinoma cells were maintained in MEM, plus phenol red supplemented with 5% (v/v) fetal bovine serum (FBS), 100 U/ml penicillin/streptomycin, 2 mM L-glutamine, and 1% (v/v) non-essential amino acids. Cells were maintained at 37°C in 5% CO2 and were routinely checked for mycoplasma. Cells were transfected using Lipofectamine 2000 (Invitrogen) and siRNA transfected using RNAiMAX (Invitrogen) according to manufacturer’s protocol and assayed 48- or 96-h after transfection, respectively.

Human granulosa lutein (hGL) cells were extracted from pooled follicular fluid, obtained at the time of egg collection for IVF/ICSI, as previously described (60, 61). The cell pellets were re-suspended in M199 (Thermo fisher Scientific, Waltham, MA USA) and layered onto a 45% Percoll (GE healthcare, Chicago, USA) gradient and then centrifuged at 1600 rpm for 30 min to separate red blood cells. The hGL cells at the interface were collected and washed with Dulbecco’s phosphate buffered saline (DPBS, Thermo Fisher Scientific). The cells were then cultured at a density of 1 x 10^5^ for 4 days in media (DMEM F-12 HAM with 10% FBS, Thermo Fisher Scientific) to allow recovery from potential effects of exogenous hormones and then cultured in serum free media for 24 h before being stored at -80°C or undergoing immediate extraction of RNA. Collection of hGL cells was approved by Hammersmith and Queen Charlotte’s Research Ethics Committee, London, UK (Reference 08/H0707/152).

### RNA Extraction and Quantitative Real-Time PCR

Total RNA was extracted using Trizol (Ambion) and the quantity and quality were evaluated using ND-2000 spectrophotometer (NanoDrop). RNA was DNase treated (Invitrogen) and converted to cDNA using Superscript First-Strand synthesis kit (Invitrogen) according to manufacturer’s protocols. mRNA was quantified using SYBR Green JumpStart Taq ReadyMix (Sigma-Aldrich) using 7900HT Fast Real-Time PCR System (Applied Biosystems). β-actin was used as housekeeping gene. Relative expression was quantified by either 2^-ΔCt^ or 2^-ΔΔCt^ method, results are displayed as raw fold change or log_2_ transformed fold change.

### Measurement of Intracellular cAMP

cAMP accumulation in cell lysates was measured using HTRF Gαs Dynamic 2 assay kit (CisBio) as per manufacturers guidance. Each condition was measured in triplicate and experiments were repeated at least three times.

### Confocal Imaging

Transfected cells were grown on glass-bottomed dishes (1.5, MatTek). On the day of imaging, cells were fed live with mouse anti-FLAG antibody for 15 min at 37°C in MEM without phenol. Cells were incubated with AlexaFluor 555 goat anti-mouse secondary antibody and basal receptor localization was imaged using a TCS-SP5 confocal microscope (Leica) with a 63X oil objective and a 1.4 numerical aperture. Cells were then treated with FSH (10 nM) for 5 min at 37°C and then re-imaged. Leica LAS AF image acquisition software was used, and images were analyzed using ImageJ.

### Measurement of Gαi/β-Arrestin Interactions *via* NanoBiT

Protein interactions in live cells, in real time, was measured using NanoLuc Binary Technology (NanoBiT, Promega) according to manufacturer’s guidance. Several DNA concentrations of SmBiT and LgBiT were tested. 48 h after transfection in a 6-well plate, cells were lifted and transfected cells from one well of a 6-well plate were aliquoted in suspension into a single well of a white flat-bottom 96-well plate (Corning). Furimazine (Promega) was added at the appropriate dilution according to manufacturer’s guidelines and basal interaction was measured for 2 min using a FLUOstar spectrofluorometer (BMG LabTech) before FSH (10 nM) was added to each well and any ligand-induced changes were measured over a period of 16 min. Measurements were taken once every 17 seconds. Basal readings were averaged and ligand-induced changes were represented as percent of basal.

### Cell Viability

The sulforhodamine B (SRB) assay was used to measure cell viability (62). Cells were plated into 96-well plates and treated as indicated in figures, replenishing treatments daily. Data are represented as raw O.D. at 510 nm, measured using a spectrophotometer (Molecular Devices).

### Lipid Droplet Measurement

Cells were plated onto coverslips, treated then fixed with 4% PFA (w/v) for 20 min at RT. Fixed cells were incubated with 5 µg/ml BODIPY 493/503 (Thermo Fisher Scientific) for 25 min at RT in the dark. Coverslips were mounted onto slides using Fluoromount G with DAPI (ThermoFisher Scientific) and cells imaged *via* confocal microscopy. Samples were imaged on a TCS SP5 (Leica) confocal microscope with 1.4 numerical aperture 63X oil-immersion objective and LAS AF image acquisition software. An excitation wavelength of 488 nm was used to visualize fluorescently labelled LDs. To quantify LD diameter and number per cell, ImageJ was used. For every cell, a line was drawn over the longest edge of each LD, the lines were measured and the diameter and number of LDs per cell was extracted from that measurement. 4-15 cells were measured per condition. Each condition was repeated in three or more independent experiments. All cells within an ROI were counted using the ImageJ plugin, Cell Counter, followed by counting of cells exhibiting BODIPY staining to calculate percentage of ‘activated’ cells.

### Bioinformatics

Human tumor gene expression data was downloaded from GDC data portal (https://portal.gdc.cancer.gov). Transcriptome data was downloaded as Fragments Per Kilobase of transcript per Million mapped reads (FPKM) format and converted to Transcripts Per Million (TPM) as TPM is more appropriate for comparing between samples (63). 0.10 TPM was the threshold for expressed protein.

### Statistics

All data unless specified otherwise represents mean ± SEM. Significance tests used included Unpaired Student’s T test when comparing means of two groups; One sample T test when comparing means with a bounded value; One- or Two-way ANOVA followed by *post-hoc* tests including Sidak’s, Bonferroni’s, and Dunnett’s tests for multiple comparisons, all using GraphPad Prism 9. Significance was considered at p<0.05. *p<0.05; **p<0.01; ***p<0.001; ****p<0.0001.

## Results

### FSH Activates Gαi/o in an Endometrial Cancer Cell Line

**Figure 1:**
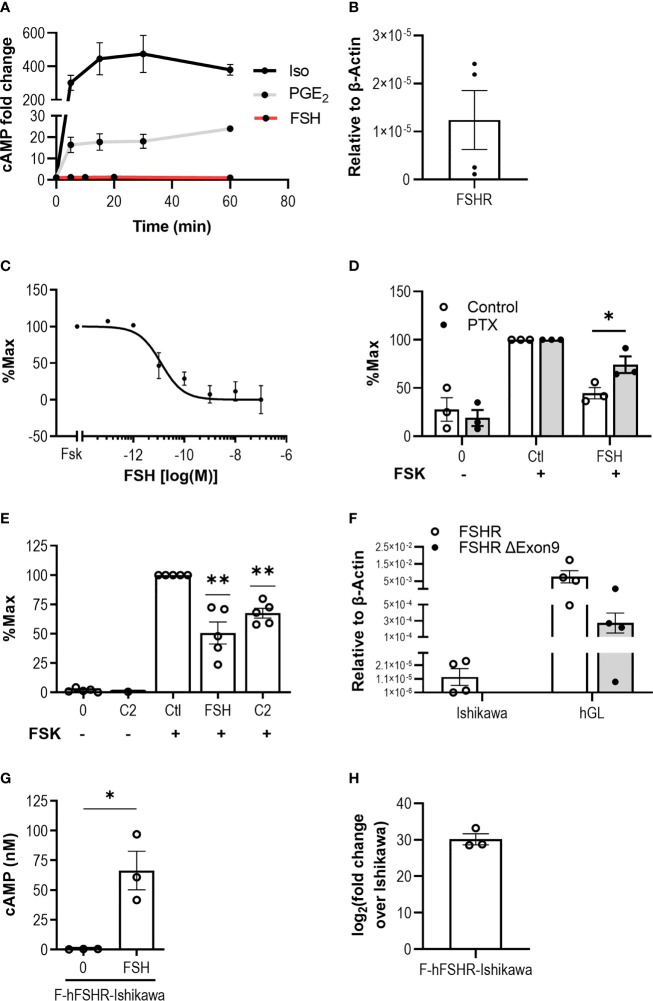
FSH activates Gαi/o in an endometrial adenocarcinoma cell line. **(A)** Ishikawa cells were treated with Isoproterenol (Iso, 10 μM) or PGE_2_ (100 nM) for 5-, 15-, 30-, or 60-min, and with FSH (10 nM) for 5-, 10-, 20-, or 60-min, following pre-treatment with IBMX (0.5 mM, 15 min). Levels of intracellular cAMP were measured *via* HTRF. Mean ± SEM, n = 3. **(B)** FSHR transcript levels in Ishikawa cells was determined using qPCR. Relative expression levels compared to housekeeper, β-Actin, was assessed using ΔCT method. Mean ± SEM, n = 4. **(C)** Levels of cAMP in response to forskolin (FSK, 3 µM) alone or with increasing concentrations of FSH (10^-13^ to 10^-7^ M) and intracellular cAMP was measured. Data presented is normalized to % FSK alone. Mean ± SEM, n = 3. **(D)** Ishikawa cells were treated with or without pertussis toxin (PTX, 500 ng/ml, 18 h) and subsequently treated with IBMX (0.5 mM, 15 min), then FSK (3 µM) alone or FSK with FSH (10 nM) for 5 min. Mean ± SEM, n = 3. Data represents % change compared to FSK alone. **(E)** Ishikawa cells were pre-treated with IBMX (0.5 mM, 15 min), then treated with or without B3 (10 μM), FSK (3 μM), FSK with FSH (10 nM), or FSK with B3 for 5 min, and intracellular cAMP was measured. Data were normalized to untreated, or to FSK alone. Mean ± SEM, n = 5. **(F)** FSHR and exon 9-deleted FSHR variant transcript expression in Ishikawa cells and primary granulosa lutein cells (hGL) was determined using qPCR. Relative expression analysis using the ΔCT method was used to assess FSHR and exon 9-deleted FSHR (FSHR ΔExon9) transcript levels compared to β-actin. Mean ± SEM, n = 4. **(G)** Ishikawa cells were transfected with human FLAG-FSHR (F-hFSHR-Ishikawa). 48 h-post transfection cells were treated with IBMX (0.5 mM, 15 min) then FSH (10 nM, 5 min), and intracellular cAMP was measured. Mean ± SEM, n = 3. **(H)** Comparison of F-hFSHR-Ishikawa FSHR transcript levels with untransfected Ishikawa. Relative expression analysis was used to assess FSHR transcript levels in F-hFSHR-Ishikawa compared to untransfected Ishikawa using the ΔΔCT method. Mean ± SEM, n = 3. Unpaired Student’s T test. *p < 0.05; **p < 0.01.

Upon activation, FSHR classically couples to Gαs in Sertoli and granulosa cells of the gonads, therefore we initially assessed the ability of FSH to activate Gαs signaling by measuring intracellular cAMP accumulation in Ishikawa cells, an epithelial endometrial adenocarcinoma cell line that expresses both estrogen and progesterone receptors (64, 65). Stimulation with FSH (10 nM) for 5-, 10-, 20- or 60-min did not induce a cAMP response over basal (**Figure 1A**). To determine whether distinct additional Gαs-coupled GPCRs could activate this pathway in this cell type, Ishikawa cells were treated with either isoproterenol (10 µM), a β-adrenergic receptor agonist, or prostaglandin E2 (PGE_2_, 100 nM), to activate EP2/EP4. Isoproterenol maximally activated Gαs to ~450-fold over basal following 30 min treatment, while PGE_2_ induced a maximal fold change of ~25-fold over basal at the 60 min timepoint, indicating that receptor-mediated activation of the Gαs/adenylate cyclase/cAMP pathway in Ishikawa cells is functional. Measurement of FSHR expression in Ishikawa cells at the mRNA level *via* quantitative real-time PCR (qPCR) indicated the receptor was expressed in these cells (**Figure 1B**).

In addition to Gαs, FSHR has been reported to couple and activate Gαi/o in both gonadal (66, 67) and extragonadal systems such as bone (30), adipose (42) and breast cancer cells (51). Therefore, we assessed whether Gαi/o-induced inhibition of adenylate cyclase could be detected in Ishikawa cells in response to FSH. Cells were stimulated with forskolin, a direct activator of adenylate cyclase, to induce intracellular cAMP accumulation. Co-treatment with FSH for 5 min was able to significantly inhibit forskolin-induced cAMP accumulation in a dose-dependent manner with an IC50 of 12.18 pM (± 5.10 SEM) (**Figure 1C**). To confirm that the reduction in intracellular cAMP was dependent on Gαi/o, cells were pre-treated with the Gαi/o inhibitor pertussis toxin (PTX). While there was no significant effect of PTX on forskolin-induced cAMP (**Supplemental Figure S1**), PTX reversed the FSH-induced negative regulation of intracellular cAMP (**Figure 1D**), indicating that FSH activates the Gαi/o pathway in this cell type. To provide support for the role of FSH-mediated activation of FSHR in these cells, an FSHR selective small molecule ligand, B3, was employed. We have previously characterized this benzamide derivative in terms of its ability to induce spatially directed cAMP signaling from the FSHR and its ability to activate multiple G protein pathways (29, 59). Ishikawa cells treated with B3 at 10 µM, a concentration we have previously demonstrated to exhibit maximal Gαi recruitment in HEK 293 cells (59), was unable to increase levels of cAMP but able to significantly inhibit forskolin-induced cAMP accumulation (**Figure 1E**), supporting the hypothesis that ligand-induced binding of FSHR activates the Gαi/o pathway in Ishikawa cells.

It has been suggested that Gαi/o-coupling in certain cell types is *via* an FSHR splice variant lacking exon 9 (38, 68, 69). Therefore, both full length FSHR and FSHR exon 9-deleted variant expression levels were measured *via* qPCR in Ishikawa cells. Primary human granulosa-lutein (hGL) cells were used as a positive control. Our results indicate that while full length FSHR was detected, albeit at low levels in Ishikawa cells, no exon 9-deleted FSHR variant transcript could be detected (**Figure 1F**). In contrast, hGLs expressed both forms of FSHR (**Figure 1F**). As FSHR mRNA levels detected in Ishikawa cells were lower than hGLs, and prior studies have demonstrated that FSHR density can alter bias between Gαs/cAMP and additional MAPK signal pathways (23), we therefore hypothesized that increasing expression of FSHR would alter its ability to activate Gαs/cAMP. To test this, we transiently over-expressed human FLAG-tagged FSHR in Ishikawa cells (F-hFSHR-Ishikawa) and measured intracellular cAMP accumulation. FSH treatment induced a significant and robust increase in intracellular cAMP levels (**Figure 1G**). FSHR overexpression relative to untransfected cells was confirmed by qPCR (**Figure 1H**). Overall, this data suggests that at a relative low receptor density, FSH/FSHR activates a Gαi/o second messenger system in Ishikawa cells.

### FSH Activation of Gαi/o Is Not Dynamin-Dependent

**Figure 2:**
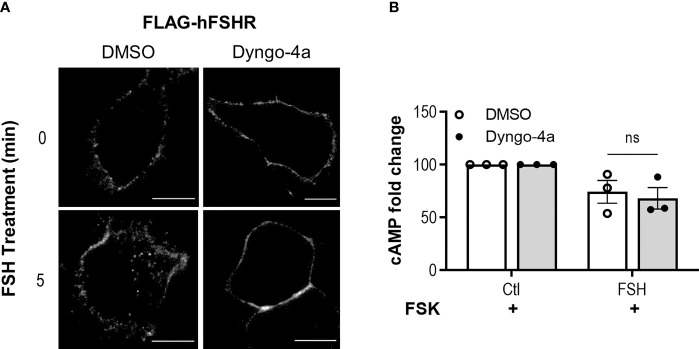
FSHR activation of Gαi/o is dynamin independent. **(A)** Ishikawa cells were transfected with human FLAG-FSHR and 48 h post transfection were pre-treated with dyngo-4a (50 µM, 40 min) or vehicle, then were fed live with anti-FLAG M1 primary antibody, labelled with alexafluor555 secondary antibody and imaged *via* confocal microscopy before (0’) or after treatment with FSH (10 nM, 5 min). Scale bar represents 10 µm. **(B)** Ishikawa cells were pretreated with dyngo-4a (50 µM, 40 min) or vehicle, then IBMX (0.5 mM, 15 min), followed by treatment with FSH (10 nM, 5 min) and intracellular cAMP was measured. Data were normalized to untreated, then relative to FSK at 100%. Data represent mean ± SEM n = 4. Unpaired Student’s T test; ns, non-significant.

We have previously demonstrated that FSH/FSHR-mediated activation of the Gαs/cAMP pathway is spatially controlled whereby dynamin-dependent receptor internalization to VEEs is essential for FSHR signaling (28, 70). We therefore determined whether inhibiting dynamin-dependent internalization altered FSH-induced Gαi/o activation in Ishikawa cells. Pre-treatment of Ishikawa cells with dyngo-4a, a potent dynamin inhibitor, inhibited FSH-induced internalization of human FLAG-FSHR expressed in Ishikawa cells, using live-cell confocal microscopy (**Figure 2A**). Unexpectedly, dyngo-4a pre-treatment did not significantly alter the ability of FSH to inhibit forskolin-induced cAMP accumulation (**Figure 2B**), suggesting that unlike Gαs, FSH-mediated activation of Gαi/o is internalization-independent.

### Chronic FSH Treatment Does Not Significantly Alter Ishikawa Cell Survival

**Figure 3:**
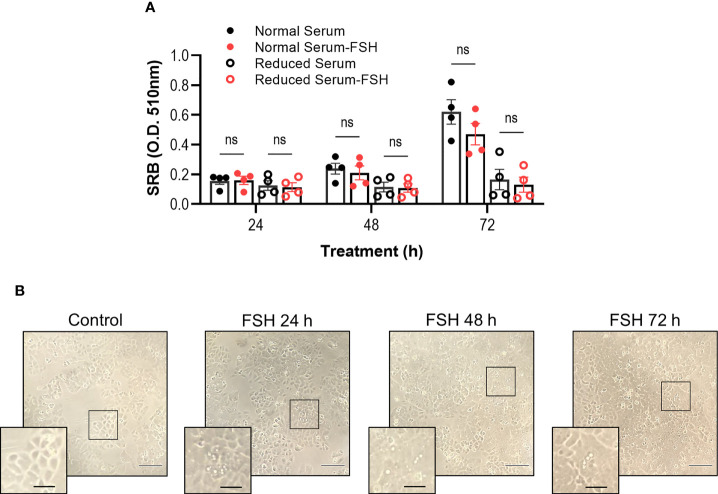
Chronic FSH treatment does not significantly alter cell viability. **(A)** Ishikawa cells grown in normal serum media (5% FBS) or reduced serum media (0.5% FBS) were treated with FSH (10 nM) for 24-, 48-, or 72-h, replenishing treatments daily, followed by the determination of cell biomass using the sulforhordamine B (SRB) assay. Raw O.D. results are displayed as mean ± SEM, n = 4. Two-way ANOVA with Tukey’s *post-hoc* test; ns, non-significant. **(B)** Representative bright field images of Ishikawa cells treated with FSH. Cells were imaged using 10X dry objective. Droplet/vesicle structures are observed in samples treated with FSH. Scale bar represents 50 μm; and scale bar in inset represents 20 μm.

Cancer cells typically rewire signaling pathways to favor pro-survival conditions (71). Previous studies have indicated a growth-promoting effect of FSH on endometrial cancer cells, HEC-1A but not Ishikawa cells (47). Given that we have identified that FSH treatment of Ishikawa cells induces a Gαi/o signaling response, we assessed whether FSH could induce growth promoting or inhibitory roles in the presence of normal or reduced-serum media following either 24-, 48-, or 72-h stimulation. FSH treatment did not significantly impact Ishikawa cell growth in either serum condition across all time points measured (**Figure 3A**). Whilst conducting these cell viability experiments, we observed in FSH treated cells an increase in intracellular droplets (**Figure 3B**). Due to these unexpected observations, we next directly assessed lipid droplet formation in this cell type.

### FSH Induces Lipid Droplet Enlargement *via* Gαi/o

To directly visualize lipid droplet (LD) formation, Ishikawa cells were incubated with a lipophilic fluorescent probe BODIPY 493/503. As a positive control for LD accumulation, Knockout Serum Replacement (KSR), a synthetic medium containing lipid-rich albumin, was used. Ishikawa cells were treated with either FSH (10 nM) or 15% KSR (v/v) for up to 72 h. Fixed and labelled cells were imaged *via* confocal microscopy to analyze both number and diameter of LDs.

**Figure 4:**
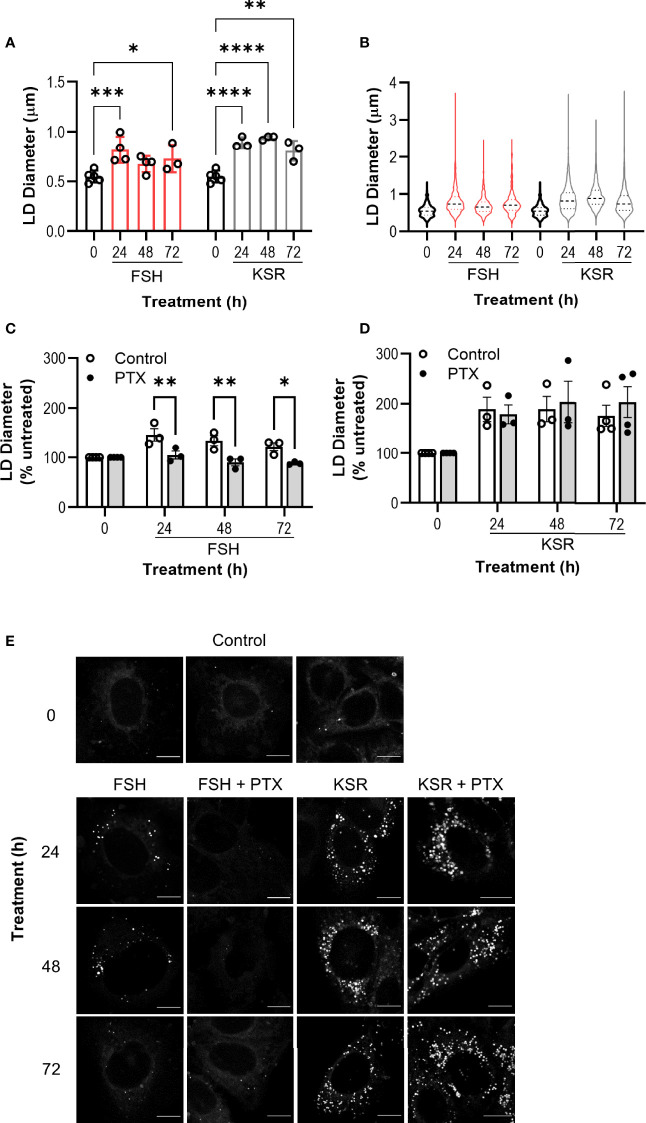
Chronic FSH induces lipid droplet enlargement *via* Gαi/o. **(A)** Ishikawa cells were treated with FSH (10 nM) or lipid-rich media, KSR (15% v/v), for 24-, 48-, or 72-h, then fixed and stained with neutral lipid dye BODIPY 493/503. Untreated (0) cells were cultured in the presence of normal serum media (5%) for 24 h. Cells were imaged using confocal microscopy and analyzed by measuring the diameter of every LD per cell using ImageJ. Minimum of 10 cells were imaged and analyzed per condition. Results represent mean LD diameter per condition ± SEM. **(B)** Violin plots of raw diameter of every LD measured in **(A)**. **(A, B)** n = 3-4. **(C, D)** Cells were pre-treated with PTX (500 ng/ml) followed by either FSH **(C)** or KSR **(D)** as in **(A)**. Data represent LD diameter normalized to control at 100%, and are shown as mean ± SEM, n = 3. Two-way ANOVA with Dunnett’s or Sidak’s *post-hoc* test. *p < 0.05; **p < 0.01; ***p < 0.001; ****p < 0.0001. **(E)** Representative images of LDs in Ishikawa cells across all treatment conditions. Scale bar represents 10 μm.

Prior to KSR or FSH treatment, BODIPY staining indicated that LDs in Ishikawa cells were either absent or if present, small in diameter and few in number (**Figures 4A, B, E** and **Supplemental Figure S2A**). Nearly all cells treated with KSR induced significant increases in both the number and the diameter at all time points (**Figures 4A, B, E** and **Supplemental Figure S2A**). While FSH did not induce a significant increase in LD number (**Supplemental Figure S2**), there was a significant increase in LD diameter to a range and average size similar to that induced by KSR, with the greatest effect seen at 24 h (**Figures 4A, B, E**). However, unlike the KSR treatment, only a subpopulation of cells induced LD formation of between ~5-25% of the field of view imaged across experiments (**Supplemental Figure S2B**).

To determine whether FSH-mediated LD enlargement was dependent on Gαi/o signaling, cells were pre-treated with PTX. PTX was able to reverse FSH-induced LD diameters at every time point measured (**Figure 4C**). Critically, PTX pretreatment had no significant effect on KSR-induced LD enlargement (**Figure 4D**).

### Involvement of β Arrestins in FSH/Gαi/o Increase in Lipid Droplet Size

**Figure 5:**
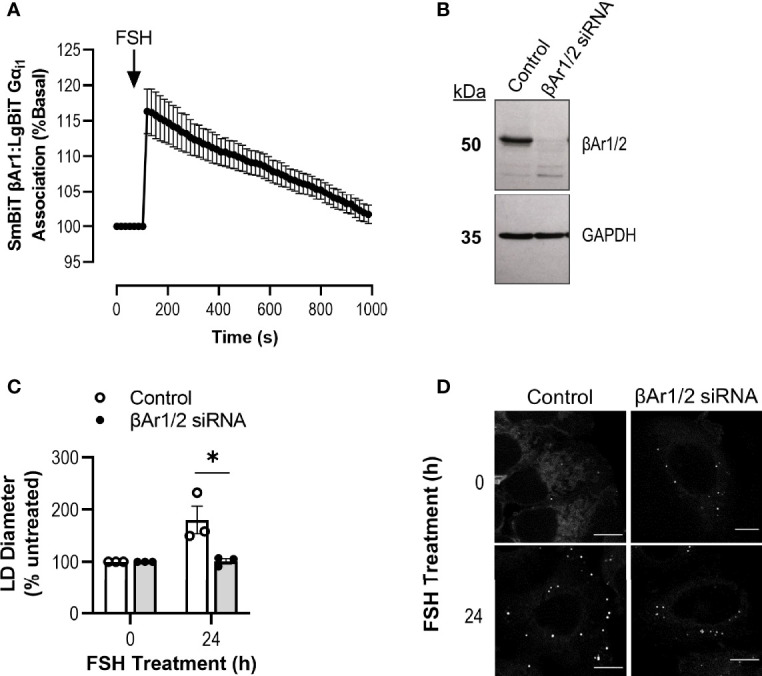
Knockdown of β-Arrestin 1/2 inhibits Gαi/o-dependent FSH action on lipid droplets. **(A)** NanoBiT live cell complementation in Ishikawa cells transfected with SmBiT βAr1 and LgBiT Gαi1 and treated with FSH (10 nM) treatment. Data represented is normalized to basal association at 100%. Data represent mean ± SEM, n = 3. **(B)** Representative western blot showing total cellular levels of β-Arrestin 1/2 following transfection with control or β-Arrestin 1/2 (βAr1/2) siRNA in Ishikawa cells. GAPDH was used as a loading control. **(C)** Cells were transfected with siRNA βAr1/2 for 96 h prior to 24 h treatment with FSH (10 nM), fixed and stained with neutral lipid dye BODIPY 493/503. Cells were imaged using confocal microscopy and analyzed by measuring the diameter of every LD per cell using ImageJ. 4-15 cells were imaged and analyzed per condition. Data were normalized to control represented as 100%. Unpaired Student’s T test was used to calculate statistical significance. Data represents mean ± SEM, n = 3. *p < 0.05. **(D)** Representative images of LDs in Ishikawa cells from **(C)** Scale bar represents 10 µm.

A recent study has identified a novel GPCR paradigm whereby association of a Gαi/o:β-arrestin 1/2 scaffold enables activation of Gαi/o signaling from GPCRs not classically coupled to Gαi/o (17). Furthermore, FSHR has been well characterized to recruit β-arrestins 1 and 2 (βAr1/2) and, pertinent to the present findings, at low receptor density has been shown to preferentially activate βAr1/2-dependent over Gαs-dependent signaling pathways (23). To test the potential involvement of a Gαi/o:βAr1/2 complex in FSH-induced effects in Ishikawa cells, the ability of FSH to enhance interactions between these two proteins was assessed *via* complementation of SmBiT-tagged βAr1 and LgBiT-tagged Gαi1 and quantified over time *via* the NanoBiT protein:protein interaction system. Luminescence from basal interactions of Gαi1 and βAr1 was rapidly increased following addition of FSH and returned to basal levels following 15 min of ligand stimulation (**Figure 5A**).

To assess the possible involvement of βAr1/2 in driving FSH-induced increases in LD diameter, βAr1/2 levels were depleted in Ishikawa cells *via* siRNA (**Figure 5B**). Cells were treated with either control or βAr1/2 siRNA, with and without FSH for 24 h, as this was the time point that induced the greatest increase in LD diameter (**Figures 4A, B**). Following knockdown of βAr1/2, FSH was unable to significantly alter LD diameter compared to control (**Figures 5C, D**). Overall, this data suggests that FSH can increase associations between Gαi1 and βAr1 and that both proteins are required for FSH-mediated LD enlargement.

### RNAseq Data From 575 Endometrial Cancer Tumors Reveals Varying Levels of FSHR, Gαi/o Subtypes, and β Arrestins

Genomic characterization of tumors has led to the identification of novel biomarkers, aided prediction of disease prognosis, and led to the identification of new disease subtypes through the discovery of specific molecular signatures. It can also be harnessed to predict patient responses to drugs and improve their safety and efficacy, also known as pharmacogenomics. The cancer genome atlas (TCGA) is a cancer genomics study that collates data of molecularly characterized human tumors from over 33 cancer types, including endometrial adenocarcinoma (72). Given our findings in the endometrial adenocarcinoma cell line, we assessed the levels of FSHR, Gαi/o family members and the β-arrestins from transcriptomic data gathered from RNA sequencing of 575 endometrial adenocarcinoma or adenoma tumors.

**Figure 6:**
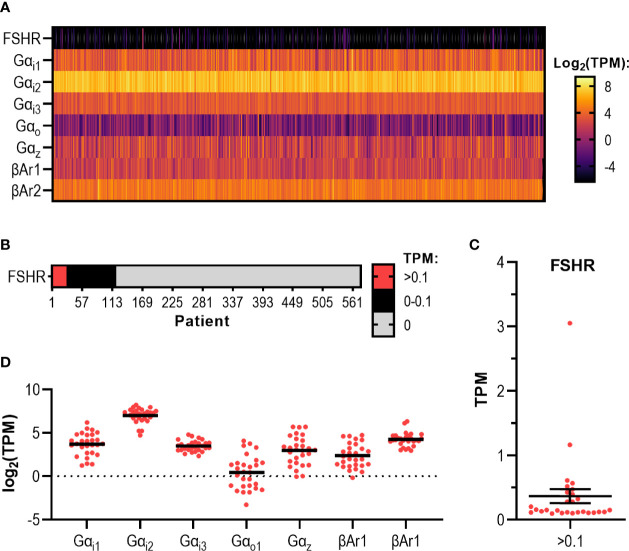
RNAseq data analysis of endometrial adenoma/adenocarcinoma tumors reveal varying levels of FSHR, Gαi subtypes, and β-arrestins. **(A)** Heatmap displaying transcript expression data of 575 endometrial adenocarcinoma/adenoma (EC) tumor samples. Results show expression levels of FSHR, Gαi/o proteins and β-arrestins and expressed in log_2_ transformed TPM, ranging from -5 to 5 log_2_ TPM. **(B)** FSHR transcript expression data for 575 EC tumors. Categorical heatmap shows patients with expression greater than 0.1 TPM (red), between 0 – 0.1 TPM (black), and 0 TPM (grey). **(C)** FSHR transcript expression over 0.1 TPM in 575 EC tumor samples analyzed by RNAseq visualized in a dot plot. **(D)** Dot plot of transcript expression data of Gαi/o proteins and β-arrestins in 28 EC tumors where FSHR transcript was expressed at a level greater than 0.1 TPM, analyzed by RNAseq.

Analysis of 575 EC patient tumors revealed that 28 patients (5%) in this cohort have FSHR expression above 0.10 transcripts per million (TPM) (**Figures 6A–C**), the presumed cut-off for when a gene is thought to be expressed as a protein in a cell (5, 73). Of this 5%, the mean expression was 0.37 (± 0.11) TPM and the maximum patient expression of FSHR was 3.05 TPM (**Figures 6B, C** and **Supplemental Figure S3**). Whilst the majority of these samples were Type 1 EC (FIGO stages I-II (74)), the sample with the highest expression was a Type 2 (stage III) (**Supplemental Figure S3**), where the cancer has spread beyond the uterus, yet still remains within the pelvic cavity (74).

These 575 samples also expressed varying levels of each Gαi/o subtype and β-arrestins (**Figure 6A**). The Gαi subtype with the highest mean expression and highest maximum expression was Gαi2 with a mean expression of 138.80 (± 2.57) TPM, with the highest expressing patient having transcript expression of 660.10 TPM (**Figure 6A**). Of the β-arrestins, βAr2 had the highest transcript level, with a mean expression of 25.34 (± 0.59) TPM and the highest expressing patient having 171.10 TPM detected (**Figure 6A**). Analysis of each Gαi/o subtype and β-arrestin specifically in the 28 samples with FSHR >0.10 TPM revealed a similar pattern whereby all genes were expressed, with Gαi2 being the highest among the Gαi/o subtypes and βAr2 higher than βAr1 (**Figure 6D**). Overall, this initial analysis of the cancer genome database indicates that a subpopulation of patients has the potential to respond to FSH-dependent mechanisms.

## Discussion

Although classically known to activate Gαs/cAMP signaling, FSH and its receptor, FSHR, can also activate additional G protein-mediated and β-arrestin-dependent pathways (22, 23, 31, 59). As demonstrated for other GPCRs, the ability of this hormone receptor system to exhibit such pleiotropic signaling has in turn opened the possibility of both ligand bias and system bias, including signal bias of disease-causing mutations or receptor variants (35, 75). Thus, the ligand, receptor and/or cellular context each provide potential to contribute to the diversity in FSH responses. Our study has supported this by identifying a cell type with low receptor levels whereby FSH primarily induces the Gαi/o pathway, associations of Gαi with a key adaptor protein for FSHR, β-arrestin, and an unexpected function in promoting LD formation. We also identified the presence of FSHR in a subgroup of patients with endometrial adenocarcinoma. Whether FSH responsiveness could represent a target and/or marker of disease progression or resistance remains to be determined, however, unveiling novel cellular actions and disease roles of gonadotropins may in turn pave the way for new therapeutic discoveries.

The inability of FSH to induce increases in cAMP in Ishikawa cells, despite the presence of a functional Gαs/cAMP pathway, confirms prior findings thought to be due to lack of FSHR expression as determined by RT-PCR (47). However, *via* qPCR, we detected low levels of full length *FSHR* transcript in the Ishikawa cell line. The ability of FSH to potently induce a Gαi/o signaling response in these cells along with a selective FSHR allosteric ligand, suggests an active FSHR present in these cells. The ability of FSHR to activate Gαi/o signaling has been reported in both gonadal (66), and non-gonadal tissues including bone, adipose, and pathophysiological contexts such as endometriotic lesions (30, 32, 40, 42) and breast cancer tissue (51). Through studies on the inactivating FSHR mutation A189V, it was demonstrated that reducing wildtype FSHR expression to a level where Gαs/cAMP is not detectable, the receptor maintains activation of MAPK pathways *via* β-arrestin-mediated signaling (23). Likewise, endogenous FSHR in a human granulosa tumor cell line also activated β-arrestin-mediated signaling without detectable Gαs signaling (76). Although Gαi/o signaling was not assessed in these studies, our results are consistent with this as the levels of FSHR in Ishikawa cells could activate, Gαi/o, while FSHR overexpression induced robust cAMP signaling. The underlying mechanism that dictates FSHR coupling under distinct expression levels is unclear at present. One possible mechanism, however, is differential spatial requirements of FSHR/G protein signaling. We have previously demonstrated that FSHR-mediated Gαs signaling is dependent on receptor internalization to VEEs that is negatively regulated by the adaptor protein APPL1 (28, 29). Unexpectedly, the FSH-mediated Gαi/o signaling in Ishikawa cells was unaffected by inhibition of receptor internalization, and thus combined with the internalization-requirement of FSHR-Gαs signaling, may suggest that altering FSHR expression levels impacts ligand-induced trafficking and the ability to activate Gαs/cAMP signaling from the correct intracellular compartment. Another possibility is the interaction of FSHR with other GPCRs as heteromers that inhibit its cAMP signaling, as recently demonstrated with FSHR and the membrane estrogen receptor GPER in human granulosa cells (27). An alternate mechanism for FSH-mediated Gαi/o signaling in non-gonadal cell systems is the presence of FSHR splice variants, however, only full length *FSHR* could be detected in Ishikawa cells.

While the presence of FSHR in extra-gonadal systems has been a subject of some controversy, this has primarily stemmed from concerns of the specificity and sensitivity of receptor antibodies employed in prior studies (44). Indeed, having widely available specific antibodies that can detect endogenously expressed GPCRs, which are often expressed at low levels *in vivo*, remains a challenge across many members of the GPCR superfamily. In our study, despite the low levels of *FSHR* mRNA detected, FSH induced a highly potent signal response (12.18 pM IC50). This was more potent than the ability of FSH to recruit Gαi *via* BRET in a HEK 293 cell system overexpressing the receptor (EC50 2.2 nM) (59). However, use of different cell types, assay readout and expression levels could contribute to these differences in potency. The development of selective FSHR small molecule allosteric ligands have highlighted the pluri-dimensionality of FSHR signaling (29, 59, 77). We employed the benzamide derivative B3 as we have previously demonstrated that this low molecular weight agonist activates distinct FSHR-signal pathways (59), including spatially controlled Gαs/cAMP signaling (29) and that this chemical class exhibits good selectivity over other glycoprotein hormone receptors (78). The ability of B3 to activate Gαi/o signaling in Ishikawa cells, supports a role for functional FSHR in this cell system. Despite the potent FSH-mediated Gαi/o signaling, our data suggests that only a subpopulation of Ishikawa cells (via BODIPY staining of LDs) may express FSHR, potentially contributing to the low levels detected by qPCR. Thus, a limitation at present is the direct biochemical detection and/or visualization of FSHR protein in these cells and future development of sensitive, selective antibodies, and potentially nanobodies, will be of high value in the field.

As highlighted above, a relationship between FSHR density and signal output has been previously drawn, whereby FSHR at low density preferentially activates β-arrestin signaling (23). In this study, we propose a requirement for β-arrestin1/2 in FSH-mediated activation of Gαi/o through complex formation in Ishikawa cells, highlighting an additional role of β-arrestins in FSHR activity. This finding is also consistent with a recent study first demonstrating that many GPCRs exhibit Gαi/o signaling *via* a Gαi/o:βAr1/2 scaffold (17). Smith et al. implicated the Gαi/o:βAr1/2 scaffold in mediating cell migration in HEK 293T cells stably expressing the angiotensin receptor, AT1R, and in primary human pulmonary artery smooth muscle cells (17). In this study, we propose a novel role for this pathway in LD enlargement, at least for FSH-mediated actions in this endometrial adenocarcinoma cell line. Although prior studies have demonstrated that FSH can induce growth in distinct endometrial cancer cell lines using hormone concentrations ranging from 100 – 3000 U/L of recombinant hormone (47), we were unable to detect any changes in cell viability in response to FSH in the Ishikawa cell line. This is in agreement with Davies et al. (47), although they were unable to detect FSHR expression in this cell type *via* RT-PCR methodology. The lack of an effect of FSH on Ishikawa cell growth, yet strong Gαi/o activation, is consistent with the finding that lower FSHR levels and inhibition of cAMP signaling ‘switches’ this receptor from apoptotic to pro-survival pathway activation (27). It is well known that to achieve the hallmarks of cancer, cancer cells rewire signaling pathways to favor pro-survival conditions (71, 79). Through these cell viability studies we serendipitously observed that cells treated with FSH appeared to have visibly large droplets/vesicles. Subsequently, we confirmed that FSH induced an increase in LD size, in a subpopulation of Ishikawa cells (5-24% depending on the ROI and across experiments), which may also account for the low levels of *FSHR* mRNA revealed by qPCR. Cancer cells are highly dynamic causing populations of cells in culture to change over time (80), which may also occur with Ishikawa cells. LD fusion and growth is regulated by the Cell death-inducing DFF45-like effector (CIDE) family of proteins that are expressed broadly across many different tissues (81–83). This LD diameter increase was shown to be PTX sensitive and dependent on βAr1/2, supporting a requirement for active Gαi/o/βAr1/2 complexes by FSH to induce LDs. LDs are highly dynamic organelles, specialized in the storage of neutral lipids, which also engage with other cellular organelles to exchange lipids and proteins. Indeed, in preadipocytes FSH increases LD formation and upregulates genes involved in lipid biosynthesis and fatty acid synthesis through a Gαi/o-dependent mechanism (32). Furthermore, free fatty acid receptor 4, a GPCR that is classically coupled to Gαq, induces LD formation *via* a Gαi/o-dependent pathway in a human hepatoma cell line (84), suggesting a broader mechanism for the GPCR/Gαi/o signaling pathway in LD regulation. For this receptor, Gαi/o-dependent LD formation can activate phosphoinositide-3 kinase, AKT and p38 MAPK signaling (84, 85), pathways FSHR is known to activate (36). It is also possible that FSHR activates crosstalk with distinct receptor systems known to drive LD formation, such as the nuclear receptor PPARgamma and/or drive changes in expression of genes related to lipid metabolism, as has been demonstrated by FSH in chicken adipose (86). Whether FSHR can activate LD formation under conditions where it is also coupled to Gαs and increases in cAMP, remains to be determined, but could be a specific feature of certain cell types, receptor levels and/or G protein activity profile.

Interestingly, LDs have been detected in many cell types, including different cancers as an adaptation of a challenging metabolic profile. Furthermore, the roles of these LDs have been implicated as a cause of neoplasia, cancer aggressiveness and a biomarker of disease recurrence and resistance to radiotherapy and chemotherapy (87–89). In the context of higher circulating lipids in EC disease state, metabolic syndrome as a risk factor for EC, and the evidence for raised lipid profiles and FSH in post-menopausal women, understanding the mechanism by which FSH causes these effects, and the role for FSH-induced LDs in endometrial cancer cells is highly pertinent and a subject of future research.

FSHR has been reported to be expressed in the endometrium (38, 39, 41, 90, 91), and aberrantly expressed in endometriosis (49, 50), a condition that increases risk of EC (92). Considering the high circulating FSH levels in premenopausal women with EC, and in postmenopausal women where EC incidence is highest, may suggest a role for FSH/FSHR in these patient groups. Human transcriptomic data acquired through next-generation sequencing can be harnessed to distinguish patient subgroups for optimal treatment. In the current study, we looked at FSHR transcript expression from RNA sequencing of 575 primary endometrial adenocarcinoma and adenoma subtype tumors. We identified a subgroup of patients with varying levels of FSHR transcript. The highest FSHR expressing tumor was Type 2/stage IIIC (3.05 TPM) EC where the tumor had metastasized to the pelvic and/or para-aortic lymph nodes (74), although most were Type 1 EC. Aberrant expression of FSHR has been linked to endometriosis, a disease which increases the risk to women of developing EC. Direct comparison of paired eutopic endometrium and ectopic lesions of patients with endometriosis found all ectopic endometrium aberrantly expressed FSHR, and half of eutopic endometrium had aberrant FSHR expression measured using qPCR. Aberrant FSHR expression and FSH action was linked to high expression of steroidogenic enzyme CYP19A and induction of vascular factors, highlighting an angiogenic potential of FSH/FSHR in endometriotic cells (49). Gαi2 and β-arrestin 2 had the highest mean expression among the patient samples with detectable *FSHR*. Gαi2 expression as well as mutations in *GNAI2* gene have been implicated in cancer cell proliferation and migration (93, 94). Prior studies have implicated β-arrestins in inhibiting GPCR-mediated apoptosis (95), including FSH/FSHR mediated apoptosis in granulosa cell tumor lines (76) and in cancer invasion and metastasis (96). With the current study suggesting additional avenues in LD regulation, the role of this functionally diverse GPCR adaptor protein may be more complex in cancer. The normal endometrium and EC are known to be highly heterogenous in cellular composition, as unveiled through the application of single cell RNA sequencing (97, 98). With the increased application of this technology, it is possible that larger patient cohorts will be studied, which in turn may lead to the identification of distinct cell types that express *FSHR*, providing additional information on the role this hormone receptor system in EC.

In summary, we have identified a cell system wherein FSHR/FSHR preferentially activates Gαi/o signaling and *via* a β-arrestin-dependent pathway, drives LD enlargement in Ishikawa cells. While the roles of these pathways in other cell types, and the impact of FSH-mediated LDs on cell function remains to be determined, it opens new avenues of investigation for EC and potential therapeutic targets, either as biomarkers that direct a stratified approach to existing therapies, or *via* exploitation of the FSH/FSHR Gαi/o-β-arrestin system.

## Data Availability Statement

The datasets presented in this study will be made available by the authors without undue reservation upon request or, can be found in online repositories. The names of the repository/repositories and accession number(s) can be found below: https://portal.gdc.cancer.gov/.

## Ethics Statement

The studies involving human participants were reviewed and approved by Hammersmith and Queen Charlotte’s Research Ethics Committee, London, UK (Reference 08/H0707/152). The patients/participants provided their written informed consent to participate in this study.

## Author Contributions

NSS performed experiments under supervision of JN, SF, and AH. HY and SSP provided the FSHR LMW compound. PA collected, cultured, and prepared hGL samples under supervision of SF. AH and NSS conceived the study and with JN, and SF designed research. NSS, PA, and AH analyzed data. NSS and AH wrote the paper. All authors contributed to the article and approved the submitted version.

## Funding

This work was supported by a Biotechnology and Biological Sciences Research Council (BBSRC) project grant (BB/S001565/1) and Genesis Research Trust awarded to AH. NS was supported by an Imperial College London President’s PhD Scholarship. PA is supported by a Medical Research Council (MRC) grant (MR/S025235/1) awarded to SF and AH.

## Conflict of Interest

HY and SP were employees of TocopheRx when sharing the compound. HY is employed by the company CanWell Pharma Inc.

The remaining authors declare that the research was conducted in the absence of any commercial or financial relationships that could be construed as a potential conflict of interest.

## Publisher’s Note

All claims expressed in this article are solely those of the authors and do not necessarily represent those of their affiliated organizations, or those of the publisher, the editors and the reviewers. Any product that may be evaluated in this article, or claim that may be made by its manufacturer, is not guaranteed or endorsed by the publisher.
