# Supplementary material for: Follicle-Stimulating Hormone Induces Lipid Droplets via Gαi/o and β-Arrestin in an Endometrial Cancer Cell Line
Source: Front Endocrinol (Lausanne). 2022 Feb 3;12:798866. doi: 10.3389/fendo.2021.798866 (PMC8850301; doi:10.3389/fendo.2021.798866)
Supplement: Supplementary file 1 [file DataSheet_1.pdf]

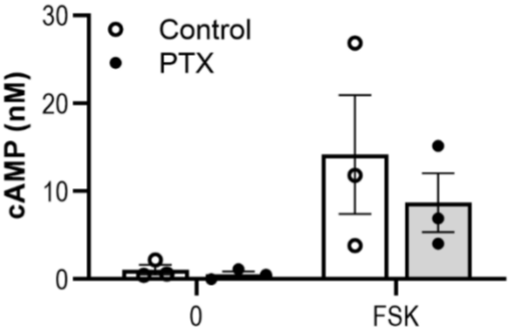

Supplementary Figure S1. Pertussis toxin treatment has no significant effect on forskolin-induced cAMP in Ishikawa cells. Ishikawa cells were treated with or without pertussis toxin (PTX, 500 ng/ml, 18 h) and subsequently treated with IBMX (0.5 mM, 15 min), then FSK (3 µM) for 5 min. Mean ± SEM, n = 3.

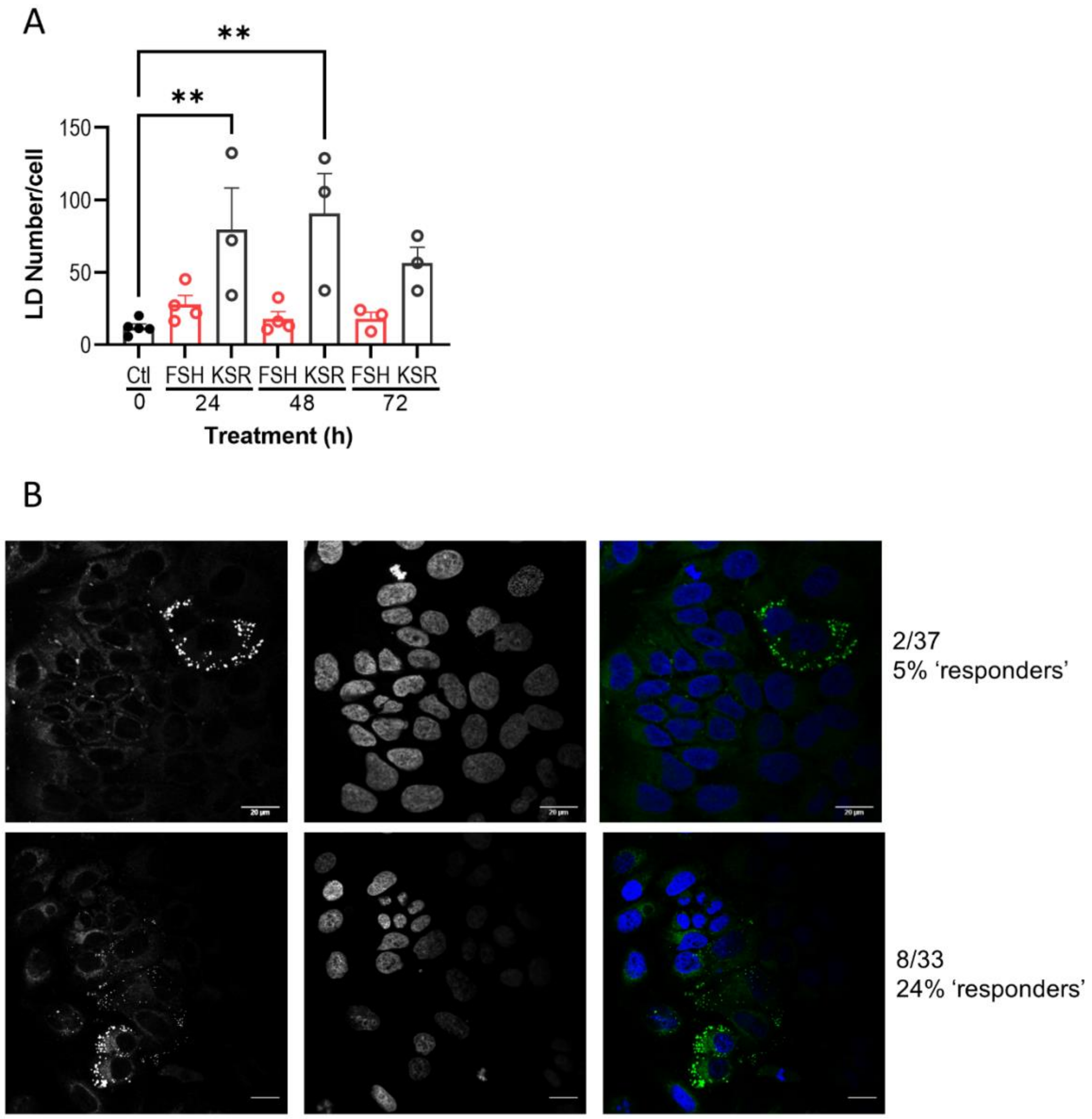

Supplementary Figure S2. FSH induces an increase in lipid droplet (LD) size but not number in a subpopulation of cells. (A) Assessment of LD number per Ishikawa cell after FSH (10 nM) or KSR (15% v/v) treatment for 24-, 48-, or 72-h. At the appropriate time point, cells were fixed and stained with neutral lipid dye BODIPY 493/503. Cells were imaged using confocal microscopy. 4-15 cells were imaged and analyzed per condition. Data represent mean ± SEM, n = 3 or more. One-way ANOVA with Dunnett’s post-hoc test, **p<0.01. **(B)** Representative images showing proportion of Ishikawa cells ‘activated’ following FSH treatment (24h) in a wider ROI. Scale bar represents 20 μm.

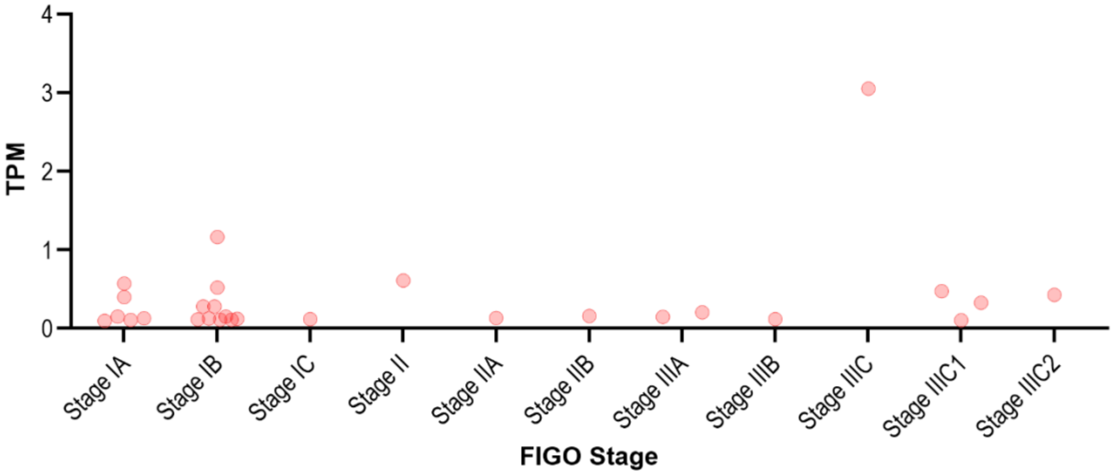

Supplementary Figure S3. FSHR expression in distinct stages of endometrial adenocarcinoma. FIGO staging of 28 human endometrial adenocarcinoma tumors expressing *FSHR* at greater than 0.10 TPM.
